# Supplementary material for: Zika Virus Infection in a Cohort of Pregnant Women with Exanthematic Disease in Manaus, Brazilian Amazon
Source: Viruses. 2020 Nov 28;12(12):1362. doi: 10.3390/v12121362 (PMC7760272; doi:10.3390/v12121362)
Supplement: Supplementary file 1 [file viruses-12-01362-s001.pdf]

Supplementary Martereials

Table S1. Characteristics of a cohort of pregnant women with exanthematic disease during the period when Zika virus transmission was most intense in Manaus, according to the results of RT-PCR ZIKV testing.

**Characteristic**	**RT-ZIKV+ (*n* = 322)**	**RT-ZIKV– (*n* = 372)**	**RT-ZIKV results indeterminate (*n* = 54)**	**RT-ZIKV under analysis (*n* = 6)**	**Not tested (*n* = 8)**
MATERNAL AGE AT INFECTION					
Maternal age (years), mean ± SD	27.0 ± 6.4	27.6 ± 6.3	27.2 ± 7.4	26.2 ± 5.7	30.3 ± 6.6
<18 years old	24 (7.5%)	23 (6.2%)	6 (11.1%)	0 (0.0%)	0 (0.0%)
18 to 29 years old	195 (60.6%)	214 (57.5%)	30 (55.6%)	5 (83.3%)	4 (50.0%)
>29 to 35 years old	77 (23.9%)	98 (26.3%)	10 (18.5%)	1 (16.7%)	2 (25.0%)
>35 years old	26 (8.1%)	37 (10.0%)	8 (14.8%)	0 (0.0%)	2 (25.0%)
MARITAL STATUS					
Single	148 (46,0%)	164 (44.1%)	28 (51.9%)	3 (50.0%)	2 (25.0%)
Married/stable union	169 (52.5%)	199 (53.5%)	25 (46.3%)	5 (50.0%)	6 (75.0%)
Separated/divorced	1 (0.3%)	7 (1.9%)	1 (1.9%)	0 (0.0%)	0 (0.0%)
Widow	0 (0.0%)	1 (0.3%)	0 (0.0%)	0 (0.0)	0 (0.0%)
Ignored	4 (1.3%)	1 (0.3%)	0 (0.0%)	0 (0.0%)	0 (0.0%)
SCHOOLING					
Without schooling	0 (0.0%)	0 (0.0%)	0 (0.0%)	0 (0.0%)	0 (0.0%)
Fundamental	49 (15.2%)	46 (12.4%)	9 (16.7%)	1 (16.7%)	1 (12.5%)
High school	185 (57.5%)	191 (51.3%)	27 (50.0%)	5 (83.3%)	3 (37.5%)
Higher education	85 (26.4%)	132 (35.5)	18 (33.3)	0 (0.0%)	4 (50.0%)
No information	3 (1.0%)	3 (0.8%)	0 (0.0%)	0 (0.0%)	0 (0.0%)

GYNECOLOGICAL HISTORY					
Parity, Mean ± SD	2.4 ± 1.5	2.3 ± 1.4	2.4 ± 1.3	2.7 ± 1.4	3.0 ± 1.4
First pregnancy No. (%)	90 (28.1)	108 (29.0)	14 (25.9)	1 (16.7)	1 (12.5)
Gestational age (weeks) at the infection					
Mean ± SD (weeks)	22.7 ± 8.9	22.2 ± 9.8	23.9 ± 9.4	17.7 ± 9.4	16.9 ± 9.1
≤22 weeks No. (%)	162 (50.3)	197 (53.0)	20 (37.0)	4 (66.7)	5 (62.5)
1st. Trimester No. (%)	62 (19.3)	97 (26.1)	10 (18.5)	2 (33.3)	3 (37.5)
2nd. Trimester No. (%)	146 (45.3)	148 (39.8)	22 (40.7)	4 (66.7)	3 (37.5)
3rd. Trimester No. (%)	114 (35.4)	127 (34.1)	22 (40.7)	0 (0.0)	2 (25.0)
ANTENATAL CARE					
Antenatal consultations at the time of the rash illness. mean ± SD	3.6 ± 0.7	3.6 ± 0.7	3.7 ± 0.5	3.2 ± 1.2	3.8 ± 0.5
Antenatal consultations at delivery, mean ± SD	7.7 ± 5.8	7.5 ± 2.5	7.6 ± 2.6	8.0 ± 2.0	7.9 ± 2.2
Birth weight, mean ± SD	3248.8 ± 575.1	330.5 ± 503.8	3212.6 ± 650.6	3540.2 ± 487.4	3306.2 ± 379.2
Pregnancy age at birth, mean ± SD (weeks)	38.7 ± 2.2	38.8 ±1.9	38.8 ± 2.3	39.3 ± 1.2	39.0 ± 1.5
CLINICAL CONDITION					
Days from the onset of symptoms to sample collection, mean ± SD	2.7 ± 3.0	3.8 ± 4.1	3.8 ± 3.6	8.0 ± 12.5	6.0 ± 3.9
Fever, No. (%)	119 (44.7)	153 (53.3)	23 (54.8)	1 (25.0)	6 (85.7)
Rash, No. (%)	273 (99.3)	275 (94.2)	48 (100.0)	5 (100.0)	5 (83.3)
Pruritus, No. (%)	249 (92.9)	256 (90.1)	47 (100.0)	4 (80.0)	5 (83.3)
Headache, No. (%)	114 (50.9)	146 (62.7)	18 (54.6)	3 (100.0)	6 (100.0)

Odynophagia, No. (%)	14 (6.7)	15 (8.0)	1 (3.2)	0 (0.0)	0 (0.0)
Eye burning, No. (%)	102 (48.6)	97 (47.6)	16 (48.5)	1 (50.0)	3 (60.0)
Ocular pruritus, No. (%)	26 (16.7)	20 (15.5)	1 (5.0)	0 (0.0)	1 (33.3)
Conjunctivitis, No. (%)	103 (49.3)	89 (44.1)	15 (48.4)	1 (50.0)	2 (40.0)
Hand arthralgia, No. (%)	142 (65.1)	124 (57.7)	18 (56.3)	1 (50.0)	1 (60)
Foot arthralgia, No. (%)	122 (56.5)	112 (53.1)	13 (41.9)	1 (50.0)	2 (40.0)
Other arthralgia, No. (%)	24 (14.8)	25 (18.1)	2 (10.0)	0 (0.0)	1 (50.0)
Hand edema, No. (%)	114 (55.3)	68 (35.6)	11 (36.7)	0 (0.0)	2 (50.0)
Foot edema, No. (%)	101 (49.5)	70 (37.0)	12 (40.0)	0 (0.0)	2 (50.0)
Asthenia/adynamia, No. (%)	79 (39.9)	82 (43.4)	17 (54.8)	0 (0.0)	2 (50.0)
Myalgia, No. (%)	106 (49.8)	126 (56.5)	15 (44.1)	0 (0.0)	4 (66.7)
Vomiting, No. (%)	34 (17.0)	55 (26.6)	3 (9.4)	0 (0.0)	1 (20.0)
Diarrhea, No. (%)	(18.8)	(19.4)	(12.1)	(50.0)	(20.0)
Lymphadenopathies, No. (%)	16 (8.3)	12 (6.8)	1 (3.6)	0 (0.0)	0 (0.0)
Vaginal bleeding, No. (%)	11 (5.5)	12 (6.3)	0 (0.0)	0 (0.0)	0 (0.0)
Other bleeding, No. (%)	14 (7.0)	14 (7.5)	2 (6.9)	0 (0.0)	0 (0.0)
Uterine contractions, No. (%)	40 (20.5)	33 (18.1)	5 (18.5)	0 (0.0)	2 (66.7)
Epidemiological contact, No. (%)	111 (69.8)	78 (58.2)	16 (76.2)	0 (0.0)	1 (33.3)
OUTCOME OF PREGNANCY, *n*	322	372	54	6	8
Intrauterine death. No. (%)	10 (3.1)	7 (1.9)	0 (0.0)	0 (0.0)	0 (0.0)
Miscarriage, No. (%)	7 (2.2)	3 (0.8)	0 (0.0)	0 (0.0)	0 (0.0)
Stillbirth, No. (%)	3 (0.9)	4 (1.1)	0 (0.0)	0 (0.0)	0 (0.0)
Live born Baby LBB No.	312	365	54	6	8
Microcephaly in LBB, No. (%)	6 (1.9)	1 (0.3)	0 (0.0)	0 (0.0)	0 (0.0)

Low birth weight, No. (%)	21 (6.7)	24 (6.6)	5 (9.3)	0 (0.0)	0 (0.0)
Preterm delivery, No. (%)	34 (10.9)	29 (8.0)	6 (11.1)	0 (0.0)	0 (0.0)

RT-PCR: reverse transcription polymerase chain reaction; ZIKV: Zika virus; SD: standard deviation; LBB: live-born baby.

Table S2. Summary of symptoms for isolated infections detected in a cohort of pregnant women who had exanthematic disease during the period when Zika virus transmission was most intense in Manaus.

Symptom	ZIKV	DENV	Syphilis	HIV	Toxoplasmosis	CMV	HERPES	PARVOV	None	Total
Eye burning	50.30%	66.67%	100.00%	0.00%	0.00%	100.00%	50.00%	37.50%	48.33%	49.41%
Hand arthralgia	67.42%	45.45%	100.00%	50.00%	66.67%	100.00%	41.18%	25.00%	63.72%	63.31%
Foot arthralgia	56.82%	45.45%	0.00%	50.00%	33.33%	100.00%	35.29%	25.00%	58.37%	55.68%
Asthenia adynamia	39.24%	27.27%	0.00%	33.33%	0.00%	100.00%	50.00%	50.00%	42.56%	41.01%
Headache	54.70%	64.29%	50.00%	50.00%	66.67%	0.00%	66.67%	62.50%	64.32%	60.43%
Conjunctivitis	52.98%	72.73%	100.00%	0.00%	50.00%	100.00%	50.00%	25.00%	44.44%	48.56%
Uterine contractions	21.79%	0.00%	0.00%	0.00%	50.00%	0.00%	18.75%	14.29%	19.89%	20.00%
Diarrhea	19.14%	10.00%	0.00%	33.33%	0.00%	0.00%	23.53%	25.00%	18.54%	18.83%
Odynophagia	4.24%	0.00%	0.00%	0.00%	0.00%	0.00%	5.88%	12.50%	7.73%	5.99%
Hand edema	54.55%	50.00%	0.00%	0.00%	0.00%	100.00%	43.75%	37.50%	36.36%	44.17%
Foot edema	49.39%	50.00%	0.00%	0.00%	0.00%	100.00%	37.50%	37.50%	38.78%	43.00%
Rash	99.14%	100.00%	100.00%	75.00%	100.00%	100.00%	100.00%	100.00%	94.70%	96.71%
Fever	47.32%	66.67%	100.00%	50.00%	33.33%	100.00%	55.56%	44.44%	55.13%	52.38%
Lymphadenopathies	6.54%	11.11%	0.00%	0.00%	0.00%	0.00%	12.50%	0.00%	4.97%	5.90%
Myalgia	50.58%	63.64%	100.00%	66.67%	33.33%	100.00%	37.50%	50.00%	58.05%	54.65%
Pruritus	93.81%	100.00%	100.00%	75.00%	66.67%	100.00%	100.00%	100.00%	90.71%	92.39%

Ocular pruritus	15.00%	11.11%	0.00%	0.00%	0.00%	0.00%	7.14%	16.67%	18.03%	15.58%
Vaginal bleeding	5.56%	0.00%	0.00%	33.33%	0.00%	0.00%	0.00%	0.00%	6.22%	5.54%
Vomiting	16.77%	30.00%	50.00%	66.67%	0.00%	0.00%	6.25%	12.50%	25.70%	21.58%

ZIKV: Zika virus; DENV: Dengue virus; CMV: cytomegalovirus; HERPES: herpes virus.
